# Evidence of Facilitation Cascade Processes as Drivers of Successional Patterns of Ecosystem Engineers at the Upper Altitudinal Limit of the Dry Puna

**DOI:** 10.1371/journal.pone.0167265

**Published:** 2016-11-30

**Authors:** Luca Malatesta, Federico Maria Tardella, Karina Piermarteri, Andrea Catorci

**Affiliations:** 1 School of Advanced Studies, University of Camerino, Camerino, Macerata, Italy; 2 School of Biosciences and Veterinary Medicine, University of Camerino, Camerino, Macerata, Italy; Estacion Experimental de Zonas Aridas, SPAIN

## Abstract

Facilitation processes constitute basic elements of vegetation dynamics in harsh systems. Recent studies in tropical alpine environments demonstrated how pioneer plant species defined as “ecosystem engineers” are capable of enhancing landscape-level richness by adding new species to the community through the modification of microhabitats, and also provided hints about the alternation of different ecosystem engineers over time. Nevertheless, most of the existing works analysed different ecosystem engineers separately, without considering the interaction of different ecosystem engineers. Focusing on the altitudinal limit of Peruvian Dry Puna vegetation, we hypothesized that positive interactions structure plant communities by facilitation cascades involving different ecosystem engineers, determining the evolution of the microhabitat patches in terms of abiotic resources and beneficiary species hosted. To analyze successional mechanisms, we used a “space-for-time” substitution to account for changes over time, and analyzed data on soil texture, composition, and temperature, facilitated species and their interaction with nurse species, and surface area of engineered patches by means of chemical analyses, indicator species analysis, and rarefaction curves. A successional process, resulting from the dynamic interaction of different ecosystem engineers, which determined a progressive amelioration of soil conditions (e.g. nitrogen and organic matter content, and temperature), was the main driver of species assemblage at the community scale, enhancing species richness. Cushion plants act as pioneers, by starting the successional processes that continue with shrubs and tussocks. Tussock grasses have sometimes been found to be capable of creating microhabitat patches independently. The dynamics of species assemblage seem to follow the nested assemblage mechanism, in which the first foundation species to colonize a habitat provides a novel substrate for colonization by other foundation species through a facilitation cascade process.

## Introduction

Facilitation processes between plant species are defined as positive plant-plant interactions in which “nurse” species create favourable microhabitats for the germination, establishment, and survival of “beneficiary” species [1], acting as a “safety net” that sustains diversity [2], by providing shelter from abiotic and biotic stresses [3]. In harsh systems, such as tropical alpine environments, these processes constitute basic elements of vegetation processes [4]. Previous studies highlighted that facilitative interactions are more common than competitive ones in conditions of high abiotic stress (i.e. stress-gradient hypothesis [5]), especially across short environmental gradients [6] or in communities composed of fewer species, under low to moderate disturbance intensities [7, 8]. Facilitation in stressful environments is particularly expected due to abiotic stress mainly induced by non-resource stressors, e.g. temperature [9], as in mountain environments [10]. Instead, facilitation is expected to be less common when abiotic stress is resource driven, as in arid environments [9, 10]. The nature and intensity of plant-plant interactions can change between apparently similar sites as a result of several factors such as: scale of analysis [11], combination of stressors [2, 7, 12, 13], variations induced by architectural or ontogenetic differences between individuals of interacting species in different populations [14, 15, 16], species-specific interaction due to the interplay between the functional features of nurse and beneficiary species [2, 12, 17, 18], relative tolerance to stress vs. competitive ability of the interacting species [9].

Positive interactions can act directly (through abiotic stress amelioration) or indirectly (e.g. competition intransitivity), but the direct modification of microhabitat is the most common form of facilitation provided by nurse species [19]. For this reason, some nurse species have been defined as “ecosystem engineers” or “foundation species” [20], as they directly transform the environment via endogenous processes that alter the structure of the engineer itself, which remains as part of the engineered environment and may be affected either positively or negatively [21]. Such species are able to modulate the availability of resources to other species and generate changes in both abiotic and biotic conditions, with the final effect of creating and maintaining microhabitat patches with a positive impact on the diversity of plant communities [21]. Recent studies focused on alpine environments at inter-tropical latitudes, demonstrated how ecosystem engineers are capable of enhancing landscape-level richness by adding new species to the community through the regulation of temperature extremes and the modification of soil properties [22]. Moreover, they demonstrated how these nurse-induced microhabitat modifications positively influenced the physiological parameters of facilitated species [23]. However, other authors found contrasting results on the effect of ecosystem engineers on species richness at a wide scale [4]. One limitation in our current understanding of the impact of engineer species on the richness of plant communities is that most of the existing works analysed different ecosystem engineers separately, or were located in areas dominated by a single nurse species. Therefore, these studies do not consider, at the scale of plant communities, the whole process of possible patch creation, alternation, coalescence and senescence, by means of the interaction of different ecosystem engineers and the degradation of microhabitat patches, that are key processes of vegetation dynamics in harsh environments [22, 24, 25]. In fact, most ecosystems are structured by multiple foundation species, whose differences in structural and functional morphology influence their impact on the community [17, 18, 26]. In addition, there are some evidences that multiple foundation species give rise to facilitation cascades, in which an independent, stress tolerant foundation species, facilitates a second, dependent foundation species to provide complementary levels of complexity and to enhance stress amelioration [20]. Facilitation cascades can drive predictable patterns in the distribution of associated organisms that tend to assemble where structural complexity and resource availability are higher [20]. The regularity with which foundation species distributions overlap suggests that emergent effects, such as facilitation cascades, may play a critical role in the organization and stabilization of many communities [27]. This calls for an integrate analysis of the temporal (i.e. dynamic succession) and spatial extent of habitat patches in tropical alpine environments [28]. Previous studies on the Peruvian Dry Puna (tropical Andes), provided hints about the interaction between ecosystem engineers at different stages of their life cycle and of a possible evolution of engineered patches due to the alternation of different engineers over time [12, 13]. To deepen our understanding of these processes, we focused our research at the upper altitudinal limit of the dry Puna vegetation, characterised by the dominance of *Festuca orthophylla* and absence of anthropic pressure, assuming higher elevations to be more stressful for plants [e.g. 7, 16] because of low air temperature, low partial pressure of CO_2_, high UV radiation, thin soils and low nutrient availability [29].

In this landscape, possible ecosystem engineering processes are due to three types of nurse species: cushions, shrubs and grass tussocks [30, 31, 32], but only a few recent studies have documented the local impact of these types of nurse species on plant diversity and community structure [30, 33, 34], even if it was widely demonstrated that in stressful environments facilitation is a key driver of species richness [35, 36]. Moreover, as regards Andean tropical alpine environments, previous studies mainly focused on the cushion species *Azorella* sp. pl. [37], but little is known about the role played by *Pycnophyllum* sp. pl., a group of cushion species that inside the dry Puna landscape is fostered by the harshest conditions [32]. Furthermore, in spite of the evidence pointing to the effects of ecosystem engineers on either species richness or species abundance, studies integrating the impacts of these effects on species diversity across different engineer species are lacking [18, 22].

We hypothesised that positive interactions structure the dry Puna community at its upper altitudinal limit, by facilitation cascades, in which the first foundation species to colonize a habitat facilitates other foundation species, and that they support diverse species assemblages, creating complex successional patterns and determining an evolution of the microhabitat associated with the patch in terms of abiotic resources and beneficiary species hosted.

Assuming patch dimension as a proxy for patch age, we used a “space-for-time” substitution to analyze successional mechanisms accounting for changes over time [38], and addressed the following research questions: i) Is there a succession between different types of ecosystem engineers in engineered patches, and does this succession follow a pattern of facilitation cascades? ii) How do different ecosystem engineers modify the microhabitat of patches? iii) How do ecosystem engineer dynamics affect species richness and composition?

## Materials and Methods

### Study area

**Fig 1:**
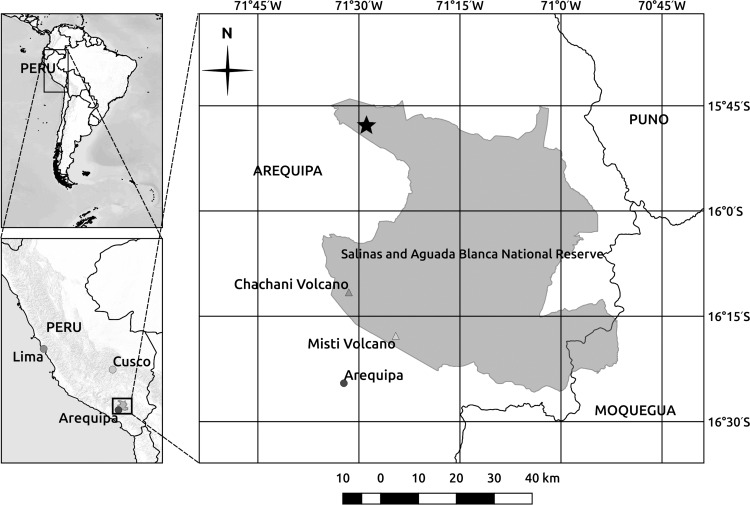
Location of the study area (indicated with a small star in the main map) within Salinas and Aguada Blanca National Reserve (Arequipa and Moquegua Departments, southern Peru)

We performed our research in the Salinas and Aguada Blanca National Reserve, in South Peru, not far from the town of Arequipa (Fig 1, central coordinates of the protected area: 16° 04’ 59” S, 71° 24’ 15” W–coordinate system: WGS84), which includes a part of the Andean Plateau and is characterised by soils with sandy texture, sub-acid pH (≈5.5) and organic matter lower than 2% [12]. Precipitation is seasonal with over 80% of the annual rainfall (300–400 mm) occurring between December and March [39]. The annual mean temperature is 3–4°C with sharp diurnal and annual variations. These tropical alpine environments are different in many aspects from the alpine temperate regions: they are characterised by higher levels of solar radiation, absence of persistent snow cover, and daily temperature oscillations that may exceed the seasonal ones and may induce daily freeze-thaw cycles. Consequently, vegetative growth occurs throughout the year, and changes in precipitation become the most important seasonal pulse, especially in drier and/or higher-elevation regions, where the climate seasonality is more marked [27].

Inside the Reserve, we chose a study area, privately owned, that comprises the typical dry Puna landscape between 4,400 and 4,600 m a.s.l. (altitudinal limit of dry Puna vegetation), dominated by cushion plants (*Pycnophyllum molle*), tussock grasses (e.g. *Festuca orthophylla* and *Calamagrostis rigida*), and resinous shrubs (*Parastrephia quadrangularis* and *P*. *lucida*). The spatial pattern of the vegetation studied has a patchy clumped structure with a matrix characterized by bare soil areas (approx. 60% cover) hosting few species with low abundance and sometimes dead wood fragments, interrupted by vegetated patches (approx. 40% cover). The human settlements are limited to small villages and isolated farms located outside of the study area, which is not used as pasture and has only been grazed by wild camelids (*Lama guanicoe* and *Vicugna vicugna*) for more than 30 years (local farmers, *pers*. *comm*.).

### Data collection

The field campaign was carried out in March 2013, during the flowering period of most plant species. The Servicio Nacional de Áreas Naturales Protegidas por el Estado (SERNANP, Peru) authorized our team to investigate in the Salinas and Aguada Blanca National Reserve. We did not collect or damage protected species during the sampling. We considered the patches engineered by each of three types of potential ecosystem engineers (hereafter EEs) present in the study area, and bare soil (non-engineered patches). We considered three types of EEs, basing on the classification of a well-recognized group of nurse plants [3, 40, 41]: cushion plants (*Pycnophyllum molle*, *P*. *weberbaueri*), grass species with tall tussock (*Festuca orthophylla*, *Calamagrostis rigida*, *C*. *heterophylla*), and shrubs higher than 40 cm (*Parastrephia lucida*, *P*. *quadrangularis*).

The study area was chosen since we aimed to deepen the very poor knowledge about the mechanism of plant assemblage at the upper altitudinal limit of dry Puna under low disturbance intensity, to test the importance of facilitative processes in tropical alpine environments regardless of the effect induced by disturbance of domestic herbivores. Therefore, using a stratified random sampling approach, we identified, through interviews with local farmers, an area of 4.5 km^2^ (central coordinates of the study site: 15° 48' 00" S, 71° 27' 50" W) that was not subjected to anthropic disturbance (e.g. mining, fires, grazing by domestic herbivores) at the altitudinal limit of dry Puna vegetation (4,400–4,600 m a.s.l.). We considered south-facing slopes because they occupied large part of the area, and selected sites with slope angles ranging from 5 to 15 degrees, excluding those with presence of latrines and/or outcropping rocks. EEs patch size was not used as stratification criterion.

Using a randomized block design, we laid 30 linear transects (blocks) parallel to contour lines, whose starting points were placed randomly using a GIS generator of random points (using the “random points” tool of QGIS software). Along each transect we laid a string, and selected the first patch for each type of EE (namely, cushion, tussock, and shrub) and bare soil intersected by the string, so that we selected along each transect a cushion, a shrub, a tussock, and an area with bare soil. We selected EE patches at a distance no less than 10 m from each other to prevent relevés from being mutually influenced. Average distance between the nearest starting points of transects was about 250 m. In each of them we recorded the nurse species, measured the maximum length (measured along the direction of maximum spread) and width (measured orthogonally to the direction of maximum length) of its canopy, and counted the individuals of each species, including seedlings of the considered nurse species. We counted individuals found inside the patch, namely inside the area occupied by a cushion or under the shrub or tussock canopy, as well as at the patch border and inside a buffer of 20 cm from the patch border. Approximating the shape of each patch to an ellipse, we calculated the area occupied by each patch by the formula S = π a b, where a and b are the semi-major axis (patch length divided by two) and the semi-minor axis (patch width divided by two), respectively. On bare soil (surface outside any engineered patch, whose living plant cover percentage did not exceed 10%, without outcropping rock cover and with possible occurrence of dead matter), we counted the individuals of each species in a circular plot of 0.5 m in radius and distant at least 1 m from the closest engineered patch. In total, we surveyed 30 patches per type of EE (namely, one patch of each type of EE per transect; 90 patches in all) and 30 plots on bare soil (namely, one plot per transect).

Since the amelioration of unfavourable conditions decreases from the canopy centre of nurse plants outwards [42, 43], for each individual of each species we recorded the respective spatial interaction type (SIT), classified as follows. SIT1, attributed to individuals growing outside the nurse canopy at a distance greater than 20 cm from the border of the nearest nurse patch (species with SIT1 can be recorded only in plots laid on bare soil). SIT2, referred to individuals growing less than 20 cm from the border of the nearest nurse patch but not under its canopy. SIT3, assigned to individuals growing in the shadow of the nurse canopy (this SIT does not apply to cushion EE species). SIT4, related to individuals growing inside the nurse canopy [13]. Valiente-Banuet and Verdú [44] defined non-facilitated species (species with SIT1 in the study case) as those recruiting more often on open ground and facilitated species (species with SITs 2, 3 and 4 in the study case) as those recruiting under or close to nurses, depending on their growth form (cushion, shrub, and tussock in the study area). With the aim of understanding the interaction and possible succession processes involving different nurse species, in each engineered patch and in plots with bare soil we recorded the number of seedlings of potential nurse species and the presence of dead matter of each type of EE (cushion, shrub, and tussock), as well as the occurrence of mature individuals of other potential nurse species in contact with the surveyed engineered patch.

In order to assess the effect of the ecosystem engineering process on microhabitats, we randomly extracted a subsample of 11 relevés carried out in each type of engineered patch and on bare soil. In each of them, we collected one soil sample (44 samples in total, 33 of which under engineered patches and 11 on bare soil). Soil samples were collected from the ground level to 20 cm depth, and analysed at the Estación Experimental Agraria—Instituto Nacional de Inovacion Agraria (INIA), water, soil, and plants analysis laboratory of Arequipa (Ministerio de Agricultura y Riego del Peru) to measure parameters related to texture (percentage of sand, loam and clay–measured with Buoyuocos' method), percentage of organic matter (modified Walkley and Black's method), percentage content of nitrogen (Micro Kjeldahl's method), potassium (flame photometry by ammonium acetate at pH 7.0) and phosphorus (modified Olsen's method 0.5 M NaHCO_3_ extraction at pH 8.8), and pH (by potentiometer at 1:2.5 soil/water suspension), following the procedures for soil analysis of the International Soil Reference and Information Centre (ISRIC), Wageningen, Netherlands [45] and the analytical methods of the Service Laboratory for soil, plant and water analysis, Royal Tropical Institute, Amsterdam [46]. In addition, to record fluctuations of soil temperature (°C), we placed three button-type electronic data loggers (iButton DS1923, Maxim Integrated Products, San Jose, CA, U.S.) at a depth of 15 cm under the centre of each type of engineered patch and on bare soil. The data loggers were set to record one value of soil temperature each two days at 2:00 PM, when the level of direct solar irradiance is maximum (we wanted to assess the effect of the nurse cover on soil features throughout the vegetative period, considering possible variations of the maximum temperature as a major factor in tropical alpine environments, since during periods of bright weather, maximum temperatures of upper soil layer are strongly influenced by the type of vegetation cover [29]). The data loggers were left in place from 15^th^ March to 15^th^ September 2013, mostly encompassing the growing period of plants and especially all phases of their reproductive cycle. We averaged data recorded by the three data loggers placed on bare soil, as well as those recorded by the three data loggers placed below each type of engineered patch. Soil temperature was available for bare soil, shrub and tussock microhabitat types.

Species nomenclature mainly followed Brako and Zarucchi [47]. We checked later taxonomic changes by consulting IPNI (http://www.ipni.org/index.html) and Tropicos (http://www.tropicos.org).

### Data analysis

#### Patch dynamics and patterns of soil variation

To test whether potential EEs at different phases of their life cycle showed preferential association with a type of EE in its mature state (namely, the nurse species engineering the surveyed patch) or with bare soil, we used an approach based on indicator species analysis (ISA). ISA is a method used to identify those items (species and species with associated SIT in the study case) that show significantly preferential distribution (in terms of frequency and abundance) in a group of samples in comparison with the other groups [48]. This method combines information on the concentration of species abundance in a particular group and the faithfulness of occurrence of a species in a particular group [49]. ISA involves the calculation of an indicator value (IV_*ij*_) for species *i* in group *j*. The IV_*ij*_ is the product of relative abundance (mean abundance of species *i* within group *j* divided by the sum of the mean abundance of species *i* in all groups) and relative frequency (number of samples in group *j* occupied by species *i* divided by the total number of samples in group *j*), and ranges from 0 to 1 [48]. Then, the group *j* in which IV_*i*_ is at its maximum is identified. Sampling units are randomly reassigned by permutations to groups a specified number of times, and each time the maximum IV_*i*_ is calculated. The probability of type I error is the proportion of times that the maximum IV_*i*_ from the randomized data set equals or exceeds the maximum IV_*i*_ from the actual data set [49]. The null hypothesis is that the maximum IV_*i*_ is no larger than would be expected by chance [49]. We tested the statistical significance (*P* < 0.05) of the observed maximum indicator values (IVs) using permutation tests with 4,999 iterations. To identify indicator species linked only to a microhabitat type, controlling for the block effect, we used permutations restricted within transects (blocks), where the microhabitat type (cushion, shrub, tussock, and bare soil) could be exchanged, but microhabitat types exchange from one transect to another was not permitted. Instead, we used unrestricted permutation tests when ISA did not involve patches inside the same transect. Only IVs higher than 0.20 were considered of interest, because values lower than 0.20 indicate species with a very low abundance and/or frequency in the data set [24].

We ran ISA on the matrices “relevés x seedlings” (number of seedlings of each EE type in relation with the surveyed patch) and “relevés x dead matter” (presence/absence of dead matter of each EE type below the surveyed patch or on bare soil), where relevés were grouped on the basis of the type of microhabitat (cushion, shrub, tussock, and bare soil). We ran another ISA on the matrix “relevés x contacts” (presence/absence of contacts between patches of each EE type and the surveyed patch), where relevés were grouped on the basis of the type of EE of the surveyed patch.

To test if the distribution of soil variables (percentage of sand, loam and clay; content of organic matter, nitrogen, phosphorus, and potassium; pH; and time series of average soil temperature) was significantly different between EEs and between EEs and bare soil, we performed Wilcoxon-Mann-Whitney tests as data did not meet the assumptions for parametric tests. We applied the Holm’s correction for multiple comparisons to reduce the likelihood of a type I error.

For the statistical analyses we used the R software (version 3.0.2 –R Foundation for Statistical Computing, Vienna, Austria http://www.R-project.org), and its stats (version 3.0–2, wilcox.test function), indicspecies (version 1.7.4, multipatt function) and permute (version 0.8–3, how function and blocks argument) packages.

#### Patterns of species richness and composition in engineered patches

To investigate the influence of the ecosystem engineering patterns on the diversity of the analysed plant community, we calculated richness for the whole set of relevés and for each group corresponding to the types of EE considered and to bare soil. We also compared the composition of the sub-communities related to the single EEs (species pools with SITs 2, 3 and 4) and to bare soil by means of the Jaccard dissimilarity index, as a measure of beta diversity, namely of the dissimilarity between communities.

To quantify the effect of all EEs on sub-community level species richness, comparing it to species richness on bare soil, we used sample-based rarefaction curves that allowed accounting for differences in sampling effort (different patch size / number of plots) within microhabitats (cushion, shrub, tussock, and bare soil) [18]. With sub-community level we refer to all the species found in relation with a type of EE and those found on bare soil. Rarefaction generates the expected number of species in a collection of n samples, drawn at random from the large pool of N samples [50]. The rarefaction curves were produced by repeatedly re-sampling the pools of N samples without replacement, in which samples are randomly accumulated in many iterations, plotting the average number of species. In addition, to quantify the effect of EEs on abundance of individuals, in comparison to bare soil, for each microhabitat we generated sample-based rarefaction curves, where the average number of individuals was plotted against the number of samples. To describe the patterns of plant-plant spatial interactions linked to the dynamics of ecosystem engineering, we executed ISAs on the “relevés x species individuals (number)” and “relevés x species individuals with associated SIT (number)” matrices, using the type of microhabitat (the three types of EE and bare soil) as grouping variable. To assess if the number of co-occurring species/individuals for each type of EE and for the overall data set were independent from the area occupied by the engineered patch, we calculated the Spearman’s correlation coefficients, as data did not meet the assumptions required for parametric tests. We also divided the relevés corresponding to each type of EE into classes defined with Sturges’ method [51] basing on the patch area. For each class we calculated descriptive statistics of the number of co-occurring species and individuals in the three subgroups corresponding to the types of EEs. To identify the indicator co-occurring species and the indicator co-occurring species with the associated SITs of each class of area in the three subgroups, we executed ISAs on the respective “relevés x co-occurring species individuals (number)” matrices and on the “relevés x co-occurring species individuals with associated SITs (number)” matrices, using surface area class as grouping variable.

For the statistical analyses we used the R software and its indicspecies (version 1.7.4, multipatt function), permute (version 0.8–3, how function and blocks argument), vegan (version 2.0–10, vegdist and diversity functions), and Hmisc (version 3.17–1, rcorr function) packages. For rarefaction analysis, we used the specaccum function of vegan package, using the “random” method and 1,000 permutations.

## Results

### Patch dynamics and patterns of soil variation

**Fig 2:**
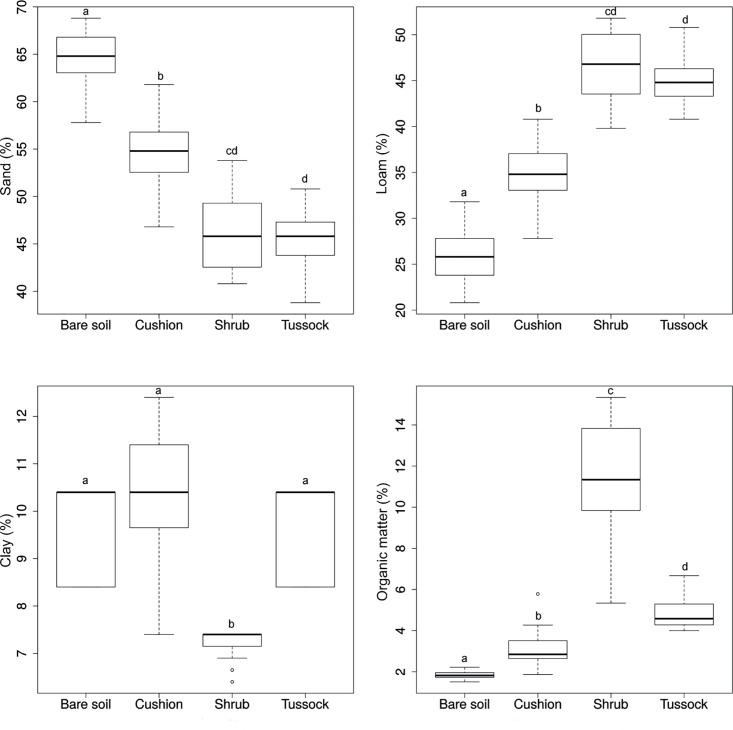
Boxplots of sand, loam, clay and organic matter content (%) of soil samples collected under the patches of ecosystem engineers and on bare soil. Equal letters indicate no statistically significant differences (*P* ≥ 0.001) in the pairwise comparisons of groups as determined by the Mann-Whitney-Wilcoxon tests, after Holm’s correction for multiple comparisons.

**Fig 3:**
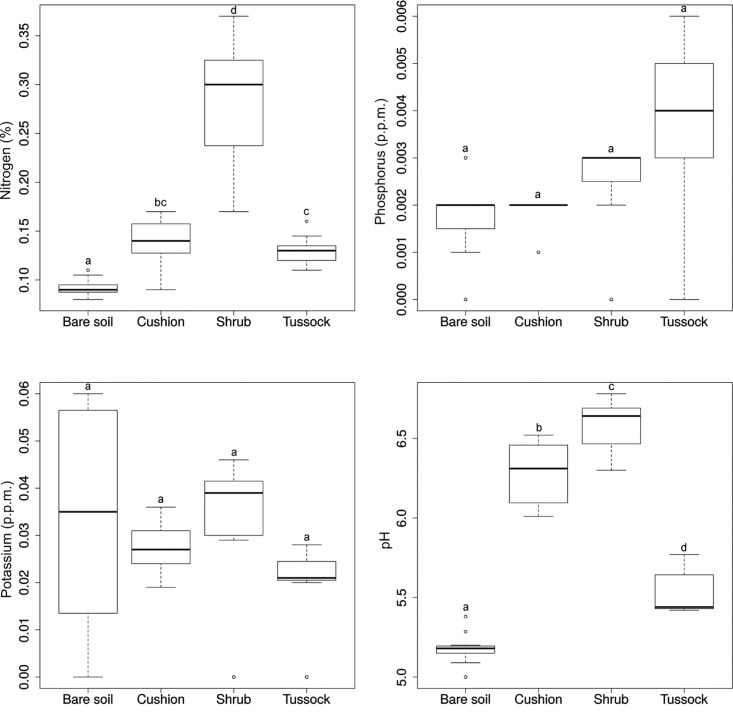
Boxplots of the chemical characteristics of soil samples collected under the patches of ecosystem engineers and on bare soil. Equal letters indicate no statistically significant differences (*P* ≥ 0.001) in the pairwise comparisons of groups as determined by the Mann-Whitney-Wilcoxon tests, after Holm’s correction for multiple comparisons.

**Fig 4:**
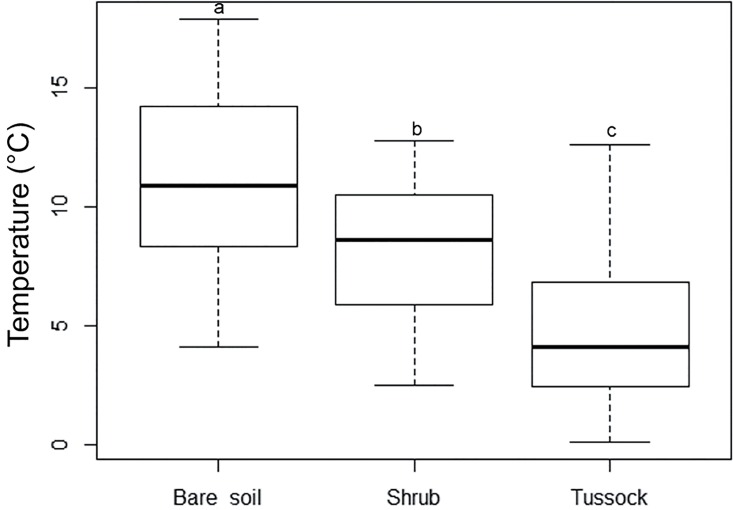
Boxplots of mean soil temperature (°C) values collected under the patches of ecosystem engineers and on bare soil. Equal letters indicate no statistically significant differences (*P* ≥ 0.001) in the pairwise comparisons of groups as determined by the Mann-Whitney-Wilcoxon tests, after Holm’s correction for multiple comparisons.

The indicator species analysis highlighted that shrub seedlings are closely associated with cushion patches (IV = 0.738, *P* < 0.001), while seedlings of tussock grass were identified as indicators for shrub patches (IV = 0.530, *P* < 0.001). We also found that cushion seedlings tend to establish on bare soil more frequently than the other types of EEs (23.3%); tussock grasses follow with 20.0%, while shrubs (3.3%) seem to have a very low ability to grow outside microhabitats provided by other EEs (data in S4 Dataset). Tussock patches were preferentially in contact with other tussocks (IV = 0.293, *P* = 0.036). With regard to the dead matter found under each type of EE and on bare soil, dead matter of cushion plants was preferentially distributed under shrub patches (IV = 0.287, *P* = 0.002), while dead matter of tussock tall grasses under tussock patches (IV = 0.289, *P* < 0.001). Bare soil had a higher mean content in coarse-grained material (sand) (64.6%) than soil beneath cushion, shrub and tussock patches (54.6, 46.2 and 45.4%, respectively) and a lower percentage of average-grain material (loam) (25.9% vs. 34.9, 46.6 and 45.1%), organic matter (1.85% vs. 3.23, 11.34, and 4.89%) and nitrogen (0.09% vs. 0.14, 0.28, and 0.13%), and lower pH (5.19 vs. 6.32, 6.61, 5.56) (Figs 2 and 3, S1 Table). Moreover, EEs appear to decrease the maximum temperature of soil, also narrowing down the fluctuation of this parameter (11.3 ± 3.4°C on bare soil; 8.1 ± 2.6°C under shrub; 4.6 ± 2.7°C under tussock) (Fig 4, S1 Table).

There were statistically significant differences (*P* < 0.001) between engineered patches and bare soil for all soil variables, except for clay percentage, K and P concentrations, while soil texture was significantly different between shrub and tussock patches as regards clay content (Figs 2–4).

### Patterns of species richness and composition in engineered patches

**Fig 5:**
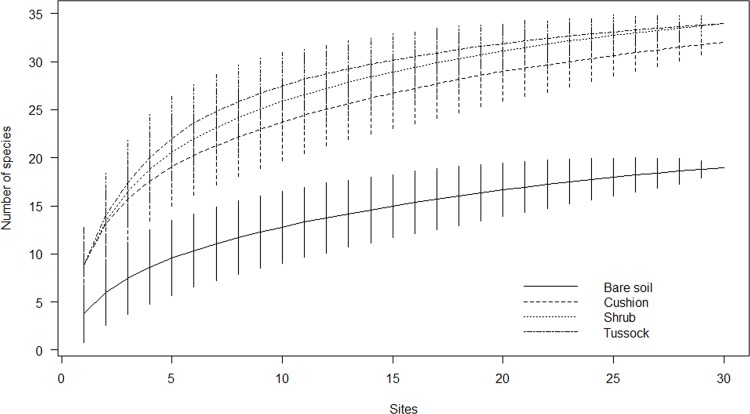
Rarefaction curves indicating the contribution of cushions, shrubs, and tussocks to community species richness in comparison with bare soil. Values are mean ± 2SD.

**Fig 6:**
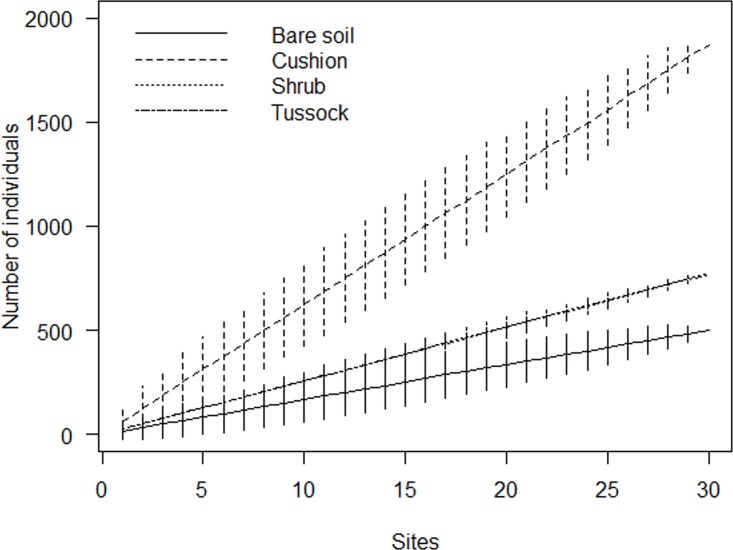
Rarefaction curves indicating the contribution of cushions, shrubs, and tussocks to abundance of individuals in comparison with bare soil. Values are mean ± 2SD.

Sample-based rarefaction curves generated from species data set did not reach an asymptote (Fig 5). Although raw species richness counts can be validly compared only when curves have reached a clear asymptote, we observed in our sample that the three EEs gave similar contributions to species richness and that their curves were higher than that of bare soil at every sample size. Plots with bare soil hosted in all 19 species out of a total pool of 45, while cushion, shrub and tussock patches had a total richness of 32, 34 and 34 species, respectively. Similarly, sample-based rarefaction curves generated using counts of individuals (average number of individuals plotted against the number of samples) did not reach any asymptote (Fig 6). Although this does not make possible to accurately compare the abundance of individuals among EEs and bare soil, each curve showed quite constant rates of increase at every sample size, up to a total number of 1,872, 776, and 771 individuals in cushion, shrub and tussock patches, respectively, and of 499 individuals on bare soil at the maximum sample size. The Jaccard dissimilarities between the sub-communities related to the microhabitat types are all above 0.50: the dissimilarities in community composition between engineered patches and bare soil increased from cushion (0.68) to shrub (0.74) to tussock (0.79) patches. The lowest dissimilarity was observed between shrub and tussock patches (0.52). The dissimilarities between cushion and shrub and between cushion and tussock were 0.74 and 0.72, respectively.

No significant correlation was identified between patch area and number of co-occurring species/individuals, neither for the single EEs nor for the whole plant community. The descriptive statistics of number of co-occurring species/individuals for the classes of patch area are reported in S2 Table (data on co-occurring species with associated SITs and patch area in S3 Dataset).

The ISA executed on the four types of microhabitat (Table 1) identified nine indicator species for cushion patches, three for shrub patches, three for tussock patches and one for plots placed on bare soil. One species of potential nurse shrub (*Parastrephia lucida*) and one of potential nurse tussock (*Festuca orthophylla*) in their mature state were among indicators of cushion patches, while two potential nurse tussocks (*Calamagrostis rigida*, *C*. *heterophylla*) and one potential nurse cushion (*Pycnophyllum molle*) in their mature state were associated with shrub patches. The only indicator species identified for plots on bare soil was *Viola granulosa*. As regards the spatial interaction types, ISA allowed to distinguish the association of the same species with different EEs due to the change in spatial interaction patterns (Table 2): for example, *F*. *orthophylla* with SITs 2 and 4 was associated with cushion patches, while the same species with SIT 3 was associated with shrub patches. In plots on bare soil, six indicator species with SIT 1 were identified. Most of the indicator species and of indicator species’ SITs were identified for medium- to large-sized patches (S3 and S4 Tables).

**Table 1:** Indicator species of the three types of ecosystem engineers and of bare soil identified by indicator species analysis performed on the “relevés x species individuals (number)” matrix, with the observed indicator value and significance level.

Group with maximum IV	Species	IV	*P*^a^
Cushion	*Calamagrostis* sp.	0.595	***
	***Parastrephia lucida***	0.416	***
	*Belloa kunthiana*	0.401	***
	*Poa aequigluma*	0.383	***
	*Nototriche turritella*	0.355	**
	***Festuca orthophylla***	0.355	***
	*Luzula racemosa*	0.316	***
	*Calamagrostis breviaristata*	0.306	*
	*Werneria aretioides*	0.276	*
Shrub	***Calamagrostis rigida***	0.500	***
	***Pycnophyllum molle***	0.281	**
	***Calamagrostis heterophylla***	0.222	**
Tussock	*Belloa longifolia*	0.677	***
	*Perezia ciliosa*	0.365	***
	*Perezia* sp.	0.306	***
Bare soil	*Viola granulosa*	0.335	*

IV, observed indicator value.

^a^ *P* is the probability of type I error, namely the proportion of times that the maximum IV_*i*_ from the randomized data set (4,999 iterations) equals or exceeds the maximum IV_*i*_ from the actual data set, under the null hypothesis that the maximum IV_*i*_ is no larger than would be expected by chance

(*P < 0.05

**P < 0.01

***P < 0.001).

Only significant indicator values (*P* < 0.05) higher than 0.20 are shown.

Potential nurse species are in bold.

**Table 2:** Indicator species with associated spatial interaction type of the three types of ecosystem engineers and of bare soil, identified by indicator species analysis performed on the “relevés x species individuals with associated SIT (number)” matrix, with the observed indicator value and significance level.

Group with maximum IV	Species	SIT^a^	IV	*P*^b^
Cushion	*Nototriche turritella*	4	0.625	***
	*Calamagrostis* sp.	4	0.600	***
	*Belloa kunthiana*	4	0.558	***
	*Calamagrostis breviaristata*	4	0.553	***
	*Poa aequigluma*	4	0.533	***
	***Parastrephia lucida***	4	0.532	***
	*Werneria aretioides*	4	0.374	***
	*Luzula racemosa*	4	0.331	***
	*Calamagrostis breviaristata*	2	0.270	**
	***Festuca orthophylla***	2	0.260	**
	***Festuca orthophylla***	4	0.257	***
Shrub	*Belloa longifolia*	4	0.343	***
	*Nototriche turritella*	3	0.321	***
	*Perezia ciliosa*	4	0.278	***
	***Calamagrostis rigida***	4	0.267	**
	***Calamagrostis rigida***	3	0.229	***
	***Calamagrostis rigida***	2	0.228	**
	*Aetheolena campanulata*	3	0.221	**
	***Festuca orthophylla***	3	0.212	**
Tussock	*Belloa longifolia*	3	0.769	***
	*Perezia ciliosa*	3	0.520	***
	*Perezia* sp.	3	0.367	***
	*Belloa kunthiana*	3	0.363	***
	*Silene andicola*	3	0.218	**
	*Gnaphalium badium*	3	0.215	***
	*Hypochaeris echegarayi*	3	0.212	**
	*Werneria aretioides*	2	0.201	*
Bare soil	*Nototriche turritella*	1	0.933	***
	*Calamagrostis breviaristata*	1	0.500	***
	*Werneria aretioides*	1	0.400	***
	*Nototriche pedicularifolia*	1	0.367	***
	*Poa aequigluma*	1	0.367	***
	*Viola granulosa*	1	0.300	***

IV, observed indicator value; SIT, spatial interaction type.

^a^ SIT1, individual growing outside the nurse canopy at a distance greater than 20 cm from the border of the nearest nurse patch; SIT2, individual growing less than 20 cm from the border of the nearest nurse patch but not under its canopy; SIT3, individual growing in the shadow of the nurse canopy; SIT4, individual growing inside the nurse canopy.

^b^ *P* is the probability of type I error, namely the proportion of times that the maximum IV_*i*_ from the randomized data set (4,999 iterations) equals or exceeds the maximum IV_*i*_ from the actual data set, under the null hypothesis that the maximum IV_*i*_ is no larger than would be expected by chance

(*P < 0.05

**P < 0.01

***P < 0.001).

Only significant indicator values (*P* < 0.05) higher than 0.20 are shown.

Potential nurse species are in bold.

## Discussion

### Patch dynamics and patterns of soil variation

We found that cushion seedlings (*Pycnophylum molle*) were able to grow on bare soil, playing a key role as foundation species [22, 24, 41], namely, as dominant species that modulate ecosystem processes having a great impact on the conditions experienced by other species [52]. Our results suggest that the maturation of seedlings of shrubs hosted as beneficiaries by cushions plants leads to the succession of new microhabitat patches, mostly formed by coalescent engineered patches, composed of large cushions (often partially dead—pers. obs.) and shrubs, instead *Festuca orthophylla* (a key nurse grass [30]) seems not in need of facilitation, since it has been observed at the border or inside the cushions, under the canopy of shrubs and on bare soil.

Beneath engineered patches several soil features were significantly ameliorated in comparison with bare soil patches, confirming the engineering role of the considered plant species. Our results are consistent with previous findings on Andean cushion species such as *Azorella monantha*, *Mulinum leptacanthum* and *Oreopolus glacialis* [53, 54], as well as *Hypericum laricifolium* shrubs [55] and *F*. *orthophylla* grass tussocks [3]. We observed a decrease of sand percentage from bare soil to shrub engineered patches, while organic matter and loam had the opposite trend. It was stated that these modifications could be related to the protection against wind erosion and water run, offered by engineering species [56, 57]. Moreover, we found that nitrogen progressively increased from bare soil to shrub-engineered patches, likely because the increase of fine-sized particles in the soil reflects in the improvement of its nutrient status [58]. Actually, we found that nitrogen progressively increased from bare soil to cushion / tussock and shrub-engineered patches. Instead, consistently with Badano et al. [22], K and P macronutrient concentrations did not show significant differences between bare soil and different types of EEs. We also observed an increase of soil pH from bare soil to shrub engineered patches. It was suggested that the ion pumping action of woody species (with deep roots), redistributes alkalinity from deeper soil layer, through the plant leaves, to the surface when leaves fall [59]. The litter accumulation, in turn, causes an increase of pH in the shallow soil layers [60]. Shrubs could trigger this process, since they function as natural barriers reducing wind velocity; this in turn leads to a deposition of wind-blown soil material that has a relatively high pH because it corresponds to soil surface material, which is the richest part of the soil profile [61]. It is noteworthy that in the study case soil pH shifts from mean values around 5.0 (bare soil) to more than 6.6 (beneath shrub), that is from unproductive to productive conditions [31, 53].

As regards soil temperature, unfortunately the malfunctioning of data loggers beneath cushions did not allow for the full understanding of the different environmental amelioration patterns related to each nurse species. However, previous research [22, 23] proved that cushion plants create thermally-buffered habitat patches with higher humidity and lower temperature than surrounding open areas. Available data about soil beneath shrubs and tussocks showed marked differences in that below the tussock patches the temperature was lower than below shrubs. This is likely due to the different canopy density (with high light irradiance *vs*. over-shading conditions). However, in both cases we observed that the mean temperatures, as well as the amplitude of their variations, were lower than on bare soil. These findings are consistent with previous studies; in fact, it was demonstrated that EEs affect the soil temperature and humidity of microhabitats, increasing the availability of water resources, limiting the drought stress and narrowing down the fluctuation of these parameters in comparison with open areas [29, 31], thus reducing the heat shock and mortality of beneficiary plants and enhancing their photosynthetic activity [53, 62].

In summary, we can argue that the observed nurse/nurse interactions besides soil amelioration and climatic mitigations, with different characteristics among engineered species, seem to highlight a successional pattern involving different types of EEs. In particular, cushion plants act as pioneers and facilitate other EEs, starting a succession process that mostly leads to shrub-dominated patches (as indicated by the presence of cushion plants dead material and by the strongest amelioration of soil conditions beneath shrubs). The dynamic role of *F*. *orthophylla* is less clear. We found that the environmental amelioration produced by *F*. *orthophylla* tussocks was less effective than that of shrubs and roughly comparable with that of *P*. *molle* cushions. Thus, it could be argued that *P*. *molle* and *F*. *orthophylla* act as two quite independent foundation species, while the encroachment of the fully developed patches (those dominated by shrubs) is facilitated by previous EEs. Therefore, it seems conceivable that a facilitation cascade process [20] is partially behind the observed dynamics. This trend can also be associated to the effects of EEs on dominance patterns. In fact, previous studies report that dominant species may become either co-dominant or subordinate with changes in the availability of resources or abiotic conditions [63], and changes in species dominance patterns have been observed for cushion nurse species in southern Chile [64].

### Patterns of species richness and composition in engineered patches

Each single type of engineered patch showed a higher species richness in comparison with plots on bare soil, as indicated by rarefaction curves. This finding confirms that, in the studied system, abiotic modulation by EEs increases species diversity by adding species that cannot survive in open areas [30, 64, 65]. This is consistent with Michalet et al. [35] and Xiao et al. [36] who assumed that facilitation in very harsh conditions has the potential to contribute to species richness at the community level because most subordinate species are positively affected by the dominant nurse species. This is also consistent with Jones et al. [21], who stated that the addition of engineered patches should almost invariably increase landscape-level species richness via a net increase in habitat diversity. In fact, in our study each type of engineered patch, characterized by a peculiar set of soil features, represents a distinct sub-community that contributes in a unique way to the overall composition of the whole plant community, as highlighted by the Jaccard dissimilarity values and by the presence of different indicator species for each EE. This result reflects the different trait composition of the considered nurse species. In fact, the interplay of nurse and beneficiary trait features plays a key role in determining the species-specific interaction [2, 13, 28] and then species assemblage. Accordingly, we found that the presence of EEs with different plant forms allows co-occurring species to differentiate their behaviour depending on the nurse species, and increases the number of available niches by providing the opportunity for various types of spatial interactions. Indeed, species may occupy different microhabitats within nurse patches due to a micro-scale environmental heterogeneity that triggers facilitation processes through within-patch niche differentiation [18, 52]. For example, species with SIT 4 are mostly associated with cushion plants, since their short, dense leaves act as a seed trap and the effects of ecosystem amelioration are maximised at the centre of their canopy [22, 41]. Conversely, tussock grasses have many species with SIT 3, due to the protection offered by their long, bent leaves that form an effective shelter from direct sunlight and herbivory [3, 30]. It is also worth noting how some species change their spatial interactions when associated with different EEs, confirming the importance of the interplay between nurse and beneficiary plant traits in determining the patterns of facilitation processes [2, 13, 28].

As regards the dimensions of microhabitat patches, even if no correlation has been found between patch area and number of co-occurring species/individuals, rarefaction curves indicated that EEs, especially tussocks and shrubs, exerted a positive effect on species richness compared to bare soil, while cushions greatly enhanced the number of individuals. Moreover, ISA highlighted that indicator co-occurring species are associated with medium- to large-sized patches, likely because of the coalescence of different engineered patches or the co-dominance of different EEs (pers. obs.). This suggests that mature engineered patches provide a key contribution to the species pool, probably because the ecosystem amelioration increases with patch dimension and age [43]. Conversely, the lower number (or absence of indicators) for the highest class of area may be due to the senescence of patches. In fact, tussocks of *F*. *orthophylla* develop in partially dead clonal garland with increase in the length but not in the width of the patch [29] and with bare inner zones, likely due to the release of toxic compounds [66]. Instead, shrubs senescence implies the death of branches and the opening of the canopy, probably triggering the start of soil erosion processes and the dropping down of environmental amelioration [55]. Moreover, palatable plants lose the protection against the herbivore bite [67].

## Conclusions

We found that a successional process, resulting from the dynamic interaction of different EEs, which determined a progressive amelioration of soil conditions, was the main driver of species assemblage at the community scale. This process seems to follow the nested assemblage mechanism, in which the first foundation species to colonize a habitat provides a novel substrate for colonization by other foundation species through facilitation cascades. In the study site, we observed that a wide set of nurse species fosters facilitative interactions within patches and dynamic interactions among different EEs. Consequently, we could infer that, since high disturbance intensity lowers the number of potential nurse species, it might thwart the facilitation cascade process among EEs, preventing soil amelioration, and decreasing availability of microhabitats and species richness. This helps understanding how anthropogenic modification of natural grazing regimes through activities, such as burning and intensive livestock grazing, can alter the dynamics among EEs, with cascading effects on dependent organisms, and why at high disturbance intensity facilitation processes have a low importance in harsh environments, indicating that sustainable management of farming systems in dry environments should prevent the loss of foundation species.

Further research is needed to understand whether the processes involved in patch dynamics, detected in the study area, are representative of the entire high Dry Puna and are relevant also at a broader scale.

## Supporting Information

S1 Table Descriptive statistics of the soil parameters of the types of ecosystem engineer and bare soil. Min., minimum; max., maximum; Qu., quartile; SD, standard deviation. Percentage of sand, loam and clay, organic matter, content of nitrogen, phosphorus, potassium, and pH were obtained from the analysis of 11 soil samples collected in each microhabitat (cushion, shrub, tussock, and bare soil); data on temperature in the soil refer to data collected using three data loggers placed at a depth of 15 cm below cushion, shrub, tussock and in bare soil.
(DOCX)

S2 Table Descriptive statistics of the number of species and individuals related to each type of ecosystem engineer for each surface area class and for the whole data set. Max., maximum; Min., minimum; Qu., quartile; SD, standard deviation Surface area classes. Cushion– 1, < 2,150 cm^2^; 2, 2,150–3,299 cm^2^; 3, 3,300–4,449 cm^2^; 4, 4,450–5,599 cm^2^; 5, 5,600–6,749 cm^2^; 6, ≥ 6,750 cm^2^. Shrub– 1, < 4,000 cm^2^; 2, 4,000–7,999 cm^2^; 3, 8,000–11,999 cm^2^; 4, 12,000–15,999 cm^2^; 5, ≥ 16,000 cm^2^. Tussock– 1, < 1,000 cm^2^; 2, 1,000–1,999 cm^2^; 3, 2,000–2,999 cm^2^; 4, 3,000–3,999 cm^2^; 5, 4,000–4,999 cm^2^; 6, ≥ 5,000 cm^2^.
(DOCX)

S3 Table Indicator species of the classes of patch area identified by indicator species analysis performed for each type of ecosystem engineer (cushion, shrub and tussock) on the “relevés x co-occurring species individuals (number)” matrix, with the observed indicator value and significance level. IV, observed indicator value. ^a^ Patch area classes. Cushion– 4, 4,450–5,599 cm^2^; 5, 5,600–6,749 cm^2^. Shrub– 4, 12,000–15,999 cm^2^; 5, ≥ 16,000 cm^2^. Tussock– 5, 4,000–4,999 cm^2^; 6, ≥ 5,000 cm^2^. ^b^ *P* value is the probability of type I error, namely the proportion of times that the maximum IV_*i*_ from the randomized data set (4,999 iterations) equals or exceeds the maximum IV_*i*_ from the actual data set, under the null hypothesis that the maximum IV_*i*_ is no larger than would be expected by chance (*P < 0.05; **P < 0.01; ***P < 0.001). Only significant indicator values (*P* < 0.05) higher than 0.20 are shown. Potential nurse species are in bold.
(DOCX)

S4 Table Indicator species with associated spatial interaction type of the classes of patch area identified by indicator species analysis performed for each type of ecosystem engineer (cushion, shrub and tussock) on the “relevés x co-occurring species individuals with associated SITs (number)” matrix, with the observed indicator value and significance level. IV, observed indicator value; SIT, spatial interaction type. ^a^ Patch area classes. Cushion– 1, < 2,150 cm^2^; 2, 2,150–3,299 cm^2^; 4, 4,450–5,599 cm^2^; 5, 5,600–6,749 cm^2^. Shrub– 4, 12,000–15,999 cm^2^; 5, ≥ 16,000 cm^2^. Tussock– 5, 4,000–4,999 cm^2^. ^b^ SIT2, individual growing less than 20 cm from the border of the nearest nurse patch but not under its canopy; SIT3, individual growing in the shadow of the nurse canopy; SIT4, individual growing inside the nurse canopy. ^c^ *P* is the probability of type I error, namely the proportion of times that the maximum IV_*i*_ from the randomized data set, based on 4,999 iterations, equals or exceeds the maximum IV_*i*_ from the actual data set, under the null hypothesis that the maximum IV_*i*_ is no larger than would be expected by chance (*P < 0.05; **P < 0.01; ***P < 0.001). Only significant indicator values (*P* < 0.05) higher than 0.20 are shown. Potential nurse species are in bold.
(DOCX)

S1 Dataset Soil parameters obtained from the analysis of 11 soil samples collected in each micro-habitat (cushion, shrub, tussock, and bare soil). (XLSX)

S2 Dataset Soil temperature measured using data loggers placed at a depth of 15 cm under cushion, shrub, tussock, and in bare soil. (XLSX)

S3 Dataset Ecosystem engineer type of the surveyed patch / bare soil, nurse species engineering each patch, patch surface area, and number of individuals of species with associated spatial interaction types. Numbers after species labels refer to spatial interaction type (1, individual growing outside the nurse canopy at a distance greater than 20 cm from the border of the nearest nurse patch; 2, individual growing less than 20 cm from the border of the nearest nurse patch but not under its canopy; 3, individual growing in the shadow of the nurse canopy; 4, individual growing inside the nurse canopy).
(XLSX)

S4 Dataset Number of seedlings of each type of potential ecosystem engineer growing on the surveyed patch and on bare soil; presence of patches of each type of potential ecosystem engineer in contact with the surveyed patch; and presence of dead matter of each type of ecosystem engineer under the surveyed patch and on bare soil. (XLSX)
