# Supplementary material for: Evidence of Facilitation Cascade Processes as Drivers of Successional Patterns of Ecosystem Engineers at the Upper Altitudinal Limit of the Dry Puna
Source: PLoS One. 2016 Nov 30;11(11):e0167265. doi: 10.1371/journal.pone.0167265 (PMC5130256; doi:10.1371/journal.pone.0167265)
Supplement: S1 Table — Min., minimum; max., maximum; Qu., quartile; SD, standard deviation. Percentage of sand, loam and clay, organic matter, content of nitrogen, phosphorus, potassium, and pH were obtained from the analysis of 11 soil samples collected in each microhabitat (cushion, shrub, tussock, and bare soil); data on temperature in the soil refer to data collected using three data loggers placed at a depth of 15 cm below cushion, shrub, tussock and in bare soil. (DOCX) [file pone.0167265.s001.docx]

S1. Table. Descriptive statistics of the soil parameters of the types of ecosystem engineer and bare soil.

|  | **Statistics** | **Sand (%)** | **Loam (%)** | **Clay (%)** | **Org. Matter (%)** | **N**  **(%)** | **P (p.p.m.)** | **K (p.p.m.)** | **pH** | **Temperature (°C)** |
| --- | --- | --- | --- | --- | --- | --- | --- | --- | --- | --- |
| Cushion | Min. | 46.8 | 27.8 | 7.4 | 1.87 | 0.09 | 0.001 | 0.019 | 6.01 | NA |
|  | 1st Qu. | 52.6 | 33.1 | 9.7 | 2.65 | 0.13 | 0.002 | 0.024 | 6.10 | NA |
|  | Mean | 54.6 | 34.9 | 10.4 | 3.23 | 0.14 | 0.002 | 0.027 | 6.32 | NA |
|  | SD | 4.0 | 3.6 | 1.5 | 1.07 | 0.02 | 0.000 | 0.005 | 0.20 | NA |
|  | Median | 54.8 | 34.8 | 10.4 | 2.85 | 0.14 | 0.002 | 0.027 | 6.31 | NA |
|  | 3rd Qu. | 56.8 | 37.1 | 11.4 | 3.51 | 0.16 | 0.002 | 0.031 | 6.46 | NA |
|  | Max. | 61.8 | 40.8 | 12.4 | 5.78 | 0.17 | 0.002 | 0.036 | 6.52 | NA |
| Shrub | Min. | 40.8 | 39.8 | 6.4 | 5.34 | 0.17 | 0.000 | 0.000 | 6.30 | 2.5 |
|  | 1st Qu. | 42.5 | 43.6 | 7.2 | 9.84 | 0.24 | 0.003 | 0.030 | 6.47 | 5.9 |
|  | Mean | 46.2 | 46.6 | 7.2 | 11.34 | 0.28 | 0.002 | 0.031 | 6.61 | 8.1 |
|  | SD | 4.5 | 4.2 | 0.4 | 3.28 | 0.07 | 0.001 | 0.016 | 0.17 | 2.6 |
|  | Median | 45.8 | 46.8 | 7.4 | 11.34 | 0.30 | 0.003 | 0.039 | 6.64 | 8.6 |
|  | 3rd Qu. | 49.3 | 50.1 | 7.4 | 13.83 | 0.33 | 0.003 | 0.042 | 6.69 | 10.5 |
|  | Max. | 53.8 | 51.8 | 7.4 | 15.34 | 0.37 | 0.003 | 0.046 | 6.78 | 12.8 |
| Tussock | Min. | 38.8 | 40.8 | 8.4 | 4.00 | 0.11 | 0.000 | 0.000 | 5.42 | 0.1 |
|  | 1st Qu. | 43.8 | 43.3 | 8.4 | 4.28 | 0.12 | 0.003 | 0.021 | 5.43 | 2.4 |
|  | Mean | 45.4 | 45.1 | 9.6 | 4.89 | 0.13 | 0.004 | 0.019 | 5.56 | 4.6 |
|  | SD | 3.5 | 2.9 | 1.0 | 0.85 | 0.01 | 0.002 | 0.010 | 0.14 | 2.7 |
|  | Median | 45.8 | 44.8 | 10.4 | 4.58 | 0.13 | 0.004 | 0.021 | 5.44 | 4.1 |
|  | 3rd Qu. | 47.3 | 46.3 | 10.4 | 5.29 | 0.14 | 0.005 | 0.025 | 5.64 | 6.8 |
|  | Max. | 50.8 | 50.8 | 10.4 | 6.67 | 0.16 | 0.006 | 0.028 | 5.77 | 12.6 |
| Bare soil | Min. | 57.8 | 20.8 | 8.4 | 1.51 | 0.08 | 0.000 | 0.000 | 5.00 | 4.1 |
|  | 1st Qu. | 63.1 | 23.8 | 8.4 | 1.74 | 0.09 | 0.002 | 0.014 | 5.15 | 8.3 |
|  | Mean | 64.6 | 25.9 | 9.6 | 1.85 | 0.09 | 0.002 | 0.033 | 5.19 | 11.3 |
|  | SD | 3.3 | 3.3 | 1.0 | 0.22 | 0.01 | 0.001 | 0.024 | 0.10 | 3.4 |
|  | Median | 64.8 | 25.8 | 10.4 | 1.83 | 0.09 | 0.002 | 0.035 | 5.18 | 10.9 |
|  | 3rd Qu. | 66.8 | 27.8 | 10.4 | 1.96 | 0.10 | 0.002 | 0.057 | 5.20 | 14.2 |
|  | Max. | 68.8 | 31.8 | 10.4 | 2.22 | 0.11 | 0.003 | 0.060 | 5.38 | 17.9 |

Min., minimum; max., maximum; Qu., quartile; SD, standard deviation.

Percentage of sand, loam and clay, organic matter, content of nitrogen, phosphorus, potassium,and pH were obtained from the analysis of 11 soil samples collected in each micro-habitat (cushion, shrub, tussock, and bare soil); data on temperature in the soil refer to data collected using three data loggers placed at a depth of 15 cm below cushion, shrub, tussock and in bare soil.
