# Supplementary material for: Evidence of Facilitation Cascade Processes as Drivers of Successional Patterns of Ecosystem Engineers at the Upper Altitudinal Limit of the Dry Puna
Source: PLoS One. 2016 Nov 30;11(11):e0167265. doi: 10.1371/journal.pone.0167265 (PMC5130256; doi:10.1371/journal.pone.0167265)
Supplement: S2 Table — Max., maximum; Min., minimum; Qu., quartile; SD, standard deviation Surface area classes. Cushion– 1, < 2,150 cm2; 2, 2,150–3,299 cm2; 3, 3,300–4,449 cm2; 4, 4,450–5,599 cm2; 5, 5,600–6,749 cm2; 6, ≥ 6,750 cm2. Shrub– 1, < 4,000 cm2; 2, 4,000–7,999 cm2; 3, 8,000–11,999 cm2; 4, 12,000–15,999 cm2; 5, ≥ 16,000 cm2. Tussock– 1, < 1,000 cm2; 2, 1,000–1,999 cm2; 3, 2,000–2,999 cm2; 4, 3,000–3,999 cm2; 5, 4,000–4,999 cm2; 6, ≥ 5,000 cm2. (DOCX) [file pone.0167265.s002.docx]

S2 Table. Descriptive statistics of the number of species and individuals related to each type of ecosystem engineer for each patch area class and for the whole data set.

| **Type of ecosystem engineer** |  |  | **Patch area class^a^** | | | | | | **Whole data set** |
| --- | --- | --- | --- | --- | --- | --- | --- | --- | --- |
|  |  |  | **1** | **2** | **3** | **4** | **5** | **6** |  |
| Cushion | Co-occurring species (no.) | Min. | 7 | 6 | 6 | 7 | 5 | 8 | 5 |
|  |  | 1st Qu. | 8 | 8 | 8 | 8 | 6 | 9 | 7 |
|  |  | Mean | 9.4 | 8.2 | 8.9 | 8.3 | 6.7 | 10.7 | 8.8 |
|  |  | SD | 1.5 | 1.9 | 2.0 | 1.5 | 1.5 | 2.3 | 2.0 |
|  |  | Median | 10 | 8 | 9 | 8 | 7 | 11 | 9 |
|  |  | 3rd Qu. | 10 | 10 | 11 | 9 | 8 | 12 | 10 |
|  |  | Max. | 11 | 11 | 12 | 10 | 8 | 12 | 12 |
|  | Individuals of co-occurring species (no.) | Min. | 35 | 10 | 37 | 97 | 13 | 43 | 10 |
|  |  | 1st Qu. | 42 | 13 | 46 | 107 | 20 | 51 | 36 |
|  |  | Mean | 60.8 | 25.7 | 68.2 | 107.7 | 32.2 | 91.3 | 62.4 |
|  |  | SD | 27.5 | 10.9 | 31.5 | 12.9 | 21.3 | 55.2 | 39.3 |
|  |  | Median | 65 | 24 | 53 | 104 | 28 | 69 | 51 |
|  |  | 3rd Qu. | 98 | 33 | 113 | 120 | 49 | 155 | 93 |
|  |  | Max. | 102 | 39 | 120 | 122 | 55 | 178 | 178 |
| Shrub | Co-occurring species (no.) | Min. | 5 | 7 | 6 | 7 | 4 |  | 5 |
|  |  | 1st Qu. | 7 | 8 | 7 | 7 | 4 |  | 8 |
|  |  | Mean | 8.6 | 9.2 | 8.3 | 8.1 | 5.6 |  | 8.7 |
|  |  | SD | 1.7 | 1.1 | 3.2 | 1.1 | 1.0 |  | 1.7 |
|  |  | Median | 8 | 9 | 7 | 8 | 5 |  | 9 |
|  |  | 3rd Qu. | 10 | 11 | 10 | 8 | 5 |  | 10 |
|  |  | Max. | 12 | 11 | 12 | 9 | 6 |  | 12 |
|  | Individuals of co-occurring species (no.) | Min. | 15 | 13 | 12 | 15 | 11 |  | 12 |
|  |  | 1st Qu. | 19 | 17 | 14 | 16 | 13 |  | 20 |
|  |  | Mean | 28.2 | 24.5 | 20.3 | 20.6 | 14.1 |  | 25.2 |
|  |  | SD | 9.1 | 7.9 | 6.6 | 1.3 | 2.3 |  | 8.4 |
|  |  | Median | 25 | 23 | 22 | 20 | 14 |  | 24 |
|  |  | 3rd Qu. | 42 | 35 | 24 | 21 | 15 |  | 28 |
|  |  | Max. | 45 | 42 | 27 | 22 | 17 |  | 45 |
| Tussock | Co-occurring species (no.) | Min. | 5 | 6 | 7 | 6 | 7 | 7 | 5 |
|  |  | 1st Qu. | 5 | 6 | 8 | 6 | 7 | 7 | 7 |
|  |  | Mean | 8.8 | 8.5 | 9.4 | 8.1 | 8.5 | 9.2 | 8.9 |
|  |  | SD | 2.5 | 1.6 | 2.3 | 2.7 | 2.2 | 2.8 | 2.0 |
|  |  | Median | 8 | 9 | 9 | 8 | 8 | 8 | 9 |
|  |  | 3rd Qu. | 9 | 10 | 11 | 9 | 9 | 9 | 10 |
|  |  | Max. | 10 | 12 | 13 | 10 | 10 | 11 | 13 |
|  | Individuals of co-occurring species (no.) | Min. | 18 | 11 | 18 | 20 | 22 | 22 | 11 |
|  |  | 1st Qu. | 20 | 16 | 23 | 23 | 25 | 24 | 18 |
|  |  | Mean | 23.5 | 21.5 | 30.1 | 27.8 | 28.4 | 26.3 | 25.6 |
|  |  | SD | 6.8 | 8.7 | 11.0 | 7.6 | 2.1 | 5.0 | 9.4 |
|  |  | Median | 22 | 18 | 28 | 28 | 29 | 25 | 24 |
|  |  | 3rd Qu. | 25 | 27 | 41 | 31 | 31 | 28 | 30 |
|  |  | Max. | 32 | 39 | 49 | 36 | 31 | 29 | 49 |

Max., maximum; Min., minimum; Qu., quartile; SD, standard deviation

^a^ Patch area classes. Cushion – 1, < 2,150 cm^2^; 2, 2,150-3,299 cm^2^; 3, 3,300-4,449 cm^2^; 4, 4,450-5,599 cm^2^; 5, 5,600- 6,749 cm^2^; 6, ≥ 6,750 cm^2^. Shrub – 1, < 4,000 cm^2^; 2, 4,000-7,999 cm^2^; 3, 8,000-11,999 cm^2^; 4, 12,000-15,999 cm^2^; 5, ≥ 16,000 cm^2^. Tussock – 1, < 1,000 cm^2^; 2, 1,000-1,999 cm^2^; 3, 2,000-2,999 cm^2^; 4, 3,000-3,999 cm^2^; 5, 4,000-4,999 cm^2^; 6, ≥ 5,000 cm^2^.
