# Supplementary material for: Evidence of Facilitation Cascade Processes as Drivers of Successional Patterns of Ecosystem Engineers at the Upper Altitudinal Limit of the Dry Puna
Source: PLoS One. 2016 Nov 30;11(11):e0167265. doi: 10.1371/journal.pone.0167265 (PMC5130256; doi:10.1371/journal.pone.0167265)
Supplement: S3 Table — IV, observed indicator value. a Patch area classes. Cushion– 4, 4,450–5,599 cm2; 5, 5,600–6,749 cm2. Shrub– 4, 12,000–15,999 cm2; 5, ≥ 16,000 cm2. Tussock– 5, 4,000–4,999 cm2; 6, ≥ 5,000 cm2. b P value is the probability of type I error, namely the proportion of times that the maximum IVi from the randomized data set (4,999 iterations) equals or exceeds the maximum IVi from the actual data set, under the null hypothesis that the maximum IVi is no larger than would be expected by chance (*P < 0.05; **P < 0.01; ***P < 0.001). Only significant indicator values (P < 0.05) higher than 0.20 are shown. Potential nurse species are in bold. (DOCX) [file pone.0167265.s003.docx]

**S3 Table. Indicator species of the classes of patch area identified by indicator species analysis performed for each type of ecosystem engineer (cushion, shrub and tussock) on the “relevés x co-occurring species individuals (number)” matrix, with the observed indicator value and significance level.**

| **Type of ecosystem engineer** | **Patch area class^a^ with maximum IV** | **Species** | **IV** | ***P*^b^** |
| --- | --- | --- | --- | --- |
| Cushion | 4 | *Calamagrostis* sp. | 0.466 | ** |
|  | 4 | ***Pycnophyllum weberbaueri*** | 0.392 | * |
|  | 4 | *Nototriche turritella* | 0.343 | * |
|  | 5 | ***Calamagrostis rigida*** | 0.404 | * |
| Shrub | 4 | *Geranium sessiliflorum* | 0.960 | * |
|  | 4 | *Draba pickeringii* | 0.830 | * |
|  | 5 | *Perezia ciliosa* | 0.843 | * |
| Tussock | 5 | *Gnaphalium badium* | 0.670 | * |
|  | 6 | ***Calamagrostis heterophylla*** | 0.568 | * |

IV, observed indicator value.

^a^ Patch area classes. Cushion – 4, 4,450-5,599 cm^2^; 5, 5,600-6,749 cm^2^. Shrub – 4, 12,000-15,999 cm^2^; 5, ≥ 16,000 cm^2^. Tussock – 5, 4,000-4,999 cm^2^; 6, ≥ 5,000 cm^2^.

^b^ *P* value is the probability of type I error, namely the proportion of times that the maximum IV*_i_* from the randomized data set (4,999 iterations) equals or exceeds the maximum IV*_i_* from the actual data set, under the null hypothesis that the maximum IV*_i_* is no larger than would be expected by chance. (**P* < 0.05; ***P* < 0.01; ****P* < 0.001).

Only significant indicator values (*P* < 0.05) higher than 0.20 are shown.

Potential nurse species are in bold.
