# Supplementary material for: Evidence of Facilitation Cascade Processes as Drivers of Successional Patterns of Ecosystem Engineers at the Upper Altitudinal Limit of the Dry Puna
Source: PLoS One. 2016 Nov 30;11(11):e0167265. doi: 10.1371/journal.pone.0167265 (PMC5130256; doi:10.1371/journal.pone.0167265)
Supplement: S4 Table — IV, observed indicator value; SIT, spatial interaction type. a Patch area classes. Cushion– 1, < 2,150 cm2; 2, 2,150–3,299 cm2; 4, 4,450–5,599 cm2; 5, 5,600–6,749 cm2. Shrub– 4, 12,000–15,999 cm2; 5, ≥ 16,000 cm2. Tussock– 5, 4,000–4,999 cm2. b SIT2, individual growing less than 20 cm from the border of the nearest nurse patch but not under its canopy; SIT3, individual growing in the shadow of the nurse canopy; SIT4, individual growing inside the nurse canopy. c P is the probability of type I error, namely the proportion of times that the maximum IVi from the randomized data set, based on 4,999 iterations, equals or exceeds the maximum IVi from the actual data set, under the null hypothesis that the maximum IVi is no larger than would be expected by chance (*P < 0.05; **P < 0.01; ***P < 0.001). Only significant indicator values (P < 0.05) higher than 0.20 are shown. Potential nurse species are in bold. (DOCX) [file pone.0167265.s004.docx]

**S4 Table. Indicator species with associated spatial interaction type of the classes of patch area identified by indicator species analysis performed for each type of ecosystem engineer (cushion, shrub and tussock) on the “relevés x co-occurring species individuals with associated SITs (number)” matrix, with the observed indicator value and significance level.**

| **Type of ecosystem engineer** | **Patch area class^a^ with maximum IV** | **Species** | **SIT^b^** | **IV** | ***P*^c^** |
| --- | --- | --- | --- | --- | --- |
| Cushion | 1 | *Poa* sp. | 4 | 0.400 | * |
|  | 2 | ***Calamagrostis rigida*** | 4 | 0.405 | * |
|  | 4 | *Calamagrostis* sp. | 4 | 0.469 | * |
|  | 4 | ***Pycnophyllum weberbaueri*** | 2 | 0.419 | * |
|  | 4 | *Nototriche turritella* | 4 | 0.344 | * |
|  | 5 | ***Calamagrostis heterophylla*** | 4 | 0.407 | * |
| Shrub | 4 | *Geranium sessiliflorum* | 4 | 0.815 | * |
|  | 4 | ***Calamagrostis heterophylla*** | 3 | 0.777 | * |
|  | 5 | *Perezia ciliosa* | 4 | 0.824 | * |
|  | 5 | *Poa aequigluma* | 4 | 0.824 | * |
| Tussock | 5 | *Senecio scorzoneraefolius* | 4 | 0.861 | * |
|  | 5 | *Gnaphalium badium* | 3 | 0.670 | * |

IV, observed indicator value; SIT, spatial interaction type.

^a^ Patch area classes. Cushion – 1, < 2,150 cm^2^; 2, 2,150-3,299 cm^2^; 4, 4,450-5,599 cm^2^; 5, 5,600-6,749 cm^2^. Shrub – 4, 12,000-15,999 cm^2^; 5, ≥ 16,000 cm^2^. Tussock – 5, 4,000-4,999 cm^2^.

^b^ SIT2, individual growing less than 20 cm from the border of the nearest nurse patch but not under its canopy; SIT3, individual growing in the shadow of the nurse canopy; SIT4, individual growing inside the nurse canopy.

^c^ *P* is the probability of type I error, namely the proportion of times that the maximum IV*_i_* from the randomized data set, based on 4,999 iterations, equals or exceeds the maximum IV*_i_* from the actual data set, under the null hypothesis that the maximum IV*_i_* is no larger than would be expected by chance

(**P* < 0.05; ***P* < 0.01; ****P* < 0.001).

Only significant indicator values (*P* < 0.05) higher than 0.20 are shown.

Potential nurse species are in bold.
